# Optimized protocol for quantification of extracellular nicotinamide adenine dinucleotide: evaluating clinical parameters and pre-analytical factors for translational research

**DOI:** 10.3389/fmed.2023.1278641

**Published:** 2024-01-08

**Authors:** Al-Hussein Ahmed Saqr, Can Kamali, Philipp Brunnbauer, Nils Haep, Pia Koch, Karl-Herbert Hillebrandt, Eriselda Keshi, Simon Moosburner, Raphael Mohr, Nathanael Raschzok, Johann Pratschke, Felix Krenzien

**Affiliations:** ^1^Department of Surgery, Campus Charité Mitte and Campus Virchow-Klinikum, Charité – Universitätsmedizin, Corporate Member of Freie Universität Berlin, Humboldt-Universität zu Berlin, and Berlin Institute of Health, Berlin, Germany; ^2^Berlin Institute of Health (BIH), Berlin, Germany; ^3^Department of Hepatology and Gastroenterology, Campus Virchow Klinikum and Campus Charité Mitte, Charité Universitätsmedizin Berlin, Berlin, Germany

**Keywords:** extracellular, nicotinamide adenine dinucleotide (NAD^+^), quantification, plasma, enzymatic assay, intracellular, clinical parameters, pre-analytical factors

## Abstract

Nicotinamide adenine dinucleotide (NAD^+^), a coenzyme for more than 500 enzymes, plays a central role in energy production, metabolism, cellular signaling, and DNA repair. Until recently, NAD^+^ was primarily considered to be an intracellular molecule (iNAD^+^), however, its extracellular species (eNAD^+^) has recently been discovered and has since been associated with a multitude of pathological conditions. Therefore, accurate quantification of eNAD^+^ in bodily fluids such as plasma is paramount to answer important research questions. In order to create a clinically meaningful and reliable quantitation method, we analyzed the relationship of cell lysis, routine clinical laboratory parameters, blood collection techniques, and pre-analytical processing steps with measured plasma eNAD^+^ concentrations. Initially, NAD^+^ levels were assessed both intracellularly and extracellularly. Intriguingly, the concentration of eNAD^+^ in plasma was found to be approximately 500 times lower than iNAD^+^ in peripheral blood mononuclear cells (0.253 ± 0.02 μM vs. 131.8 ± 27.4 μM, *p* = 0.007, respectively). This stark contrast suggests that cellular damage or cell lysis could potentially affect the levels of eNAD^+^ in plasma. However, systemic lactate dehydrogenase in patient plasma, a marker of cell damage, did not significantly correlate with eNAD^+^ (*n* = 33; *r* = −0.397; *p* = 0.102). Furthermore, eNAD^+^ was negatively correlated with increasing c-reactive protein (CRP, *n* = 33; *r* = −0.451; *p* = 0.020), while eNAD^+^ was positively correlated with increasing hemoglobin (*n* = 33; *r* = 0.482; *p* = 0.005). Next, variations in blood drawing, sample handling and pre-analytical processes were examined. Sample storage durations at 4°C (0–120 min), temperature (0° to 25°C), cannula sizes for blood collection and tourniquet times (0 – 120 s) had no statistically significant effect on eNAD^+^ (*p* > 0.05). On the other hand, prolonged centrifugation (> 5 min) and a faster braking mode of the centrifuge rotor (< 4 min) resulted in a significant decrease in eNAD^+^ levels (*p* < 0.05). Taken together, CRP and hemoglobin appeared to be mildly correlated with eNAD^+^ levels whereas cell damage was not correlated significantly to eNAD^+^ levels. The blood drawing trial did not show any influence on eNAD^+^, in contrast, the preanalytical steps need to be standardized for accurate eNAD^+^ measurement. This work paves the way towards robust eNAD^+^ measurements, for use in future clinical and translational research, and provides an optimized hands-on protocol for reliable eNAD^+^ quantification in plasma.

## 1 Introduction

Nicotinamide adenine dinucleotide (NAD^+^) is a coenzyme of utmost importance in many biological processes, playing a crucial role in regulating metabolism, cellular signaling, energy production as well as DNA repair. Recent research highlighted its involvement and potential therapeutic role in mitochondrial and age-related disorders (1, 2), cardiovascular disease (3, 4), neurodegenerative disease (1), and metabolic disorders (5, 6), wherein pathological changes and the onset of such diseases were usually predicated or associated with a dysregulation of bodily NAD^+^ levels.

As of June 2023, there were approximately 1,000 interventional clinical trials on NAD^+^ or its precursors listed on clinicaltrials.gov, assessing their efficacy as therapeutic agents. Yet, most of these trials merely focus on measuring a clinical outcome with respect to NAD^+^ precursor substitution therapy or simply evaluate the safety profile of NAD^+^ precursors. In order to take these trials beyond safety evaluation and to unlock pharmacokinetic and -dynamic insights, which enable the development of dose–response models and thereby therapeutic guidelines for NAD^+^ precursor substitution therapy, the quantification of NAD^+^ levels in bodily fluids is imperative.

Until recently, NAD^+^ was primarily considered to be an intracellular molecule (iNAD^+^) exclusively, however, its extracellular species (eNAD^+^) has recently been discovered and has since been associated with a multitude of pathological conditions (7). eNAD^+^ levels are supposed to change over time due to metabolic activities and pathological conditions such as injury, inflammation, and infection (8). NAD^+^ can flux via Connexin43 hemichannels or as a result of apoptosis, from intracellular to the extracellular space (9). Moreover, eNAD^+^ can be metabolized via ectoenzymes such as CD38, CD73, nucleotide pyrophosphatase/phosphodiesterase 1 and ADP-ribsoyltransferases (ARTS) on the cell membrane (9).

To date, there are two widely accepted methods for NAD^+^ quantification in plasma, high performance liquid chromatography tandem mass spectrometry (HPLC/MS/MS) (10) and enzymatic cycling – also known as reactant recycling – assays (10, 11). Despite their widespread use, HPLC/MS/MS have a high method complexity and are prone to difficulties distinguishing metabolites due to pre-analytical processes (12). On the other hand, enzymatic assays are simpler, cost-effective, and highly reproducible (11).

In order for NAD^+^ to become a clinically relevant metabolite and achieve biomarker significance, the influence of variations in pre-analytical processes on NAD^+^ concentrations must be evaluated to correct for the presence of potential confounders. While many studies have characterized the influence of pre-analytical process variations on several routinely assayed metabolites such as glucose, creatinine, acylcarnitines, urea, uric acid, ammonia, bilirubin, bile acids, cholesterol, amino acids and fatty acids (13–16), no reports exist on the effects of pre-analytical method variations on eNAD^+^ quantification.

Since the impact of variations in routine clinical laboratory values and experimental processing steps on eNAD^+^ remains unknown, this study examines the influence of blood parameters, variations in sample collection, handling and pre-analytical steps on measured eNAD^+^ levels in human plasma. This study not only provides substantial evidence but also introduces the first practical protocol for handling blood samples with precision in measuring eNAD^+^, paving the way for future clinical research on NAD^+^.

## 2 Materials and methods

### 2.1 Statistical hypothesis

H0: eNAD^+^ concentrations in human heparinized plasma are significantly affected by blood parameters and/or variations in sample collection methods and/or pre-analytical processing steps including cannula size, tourniquet duration, storage conditions (time and temperature), and/or centrifugation parameters (speed, duration, deceleration mode).

H1: eNAD^+^ concentrations in human heparinized plasma are not significantly affected by blood parameters and/or variations in sample collection methods and/or pre-analytical processing steps including cannula size, tourniquet duration, storage conditions (time and temperature), and/or centrifugation parameters (speed, duration, deceleration mode).

### 2.2 Patient cohort

The study was approved by the Charité ethics committee (Ethikkommission der Charité Universitätsmedizin Berlin, EA2/159/22, EA1/291/16, and EA1/096/18) in compliance with the local regulatory guidelines, the Declaration of Helsinki. The data (*n* = 38) was gathered from both a healthy cohort (*n* = 5) and a disease cohort (*n* = 33) to analyze potential correlations in eNAD^+^ levels in response to changes in blood parameters. Patients have been recruited between August 2022 and March 2023. Informed consent was collected from all participants prior to any study related procedures.

#### 2.2.1 Healthy cohort

Healthy male and female volunteers were recruited who met the inclusion age of 18–65 years. Participants suffering from acute or chronic disease, ongoing pregnancy, or taking regular medications were excluded from the cohort. A total of ten healthy volunteers including two women and eight men with a mean age of 31.4 ± 5.4 (mean ± standard deviation) years were recruited in the study. After a 12 h fasting period, blood was collected into lithium heparin tubes (BD Vacutainer Heparin Tubes, catalog number: 367886) by means of venipuncture.

#### 2.2.2 Disease cohort

Additionally, 33 patients with a mean age of 64.1 ± 16.8 have been recruited with the following eligibility criteria: age ≥ 18 male or female, hepatocellular carcinoma, pancreas cancer, colorectal carcinoma, cholangiocarcinoma, and liver cirrhosis. Patients did not have surgery nor any adjuvant treatment before blood collection. Patients who did not give informed consent have been excluded.

### 2.3 Clinical laboratory parameters

To investigate potential correlations between eNAD^+^, clinical blood parameters were measured by a specialist laboratory (Labor Berlin – Charité Vivantes GmbH, Germany).

### 2.4 Peripheral blood mononuclear cell isolation

Freshly drawn blood samples from healthy donors and patients were collected in 9 mL lithium heparin tubes (BD Vacutainer Heparin Tubes, catalog number: 367886) using a 21 G cannula (Braun, Venofix A, Catalog No: 4056504-01) with 30 s of tourniquet time. PBMCs isolation was carried out in SepMate-50 tubes (StemCell Technologies, Catalog No: 85450) according to the manufacturer’s instructions. After isolation, PBMCs were counted using a CASY cell counter (Omni Life Science, Catalog No: 5651736) and 5 × 10^6^ cells were pelleted for iNAD^+^ extraction and quantification.

### 2.5 eNAD^+^ enzymatic assay

Blood was collected into lithium heparin tubes (BD Vacutainer Heparin Tubes, Catalog No: 367886) using a 21 G cannula (Braun, Venofix A, Catalog No: 4056504-01) with 30 s of tourniquet time. Sample processing was carried out after blood collection, starting with centrifugation at 2500 g for 15 min at 4°C to separate corpuscular blood parts from plasma (11). Plasma was then aliquoted, snap frozen, and stored at −80°C. Samples were thawed at room temperature followed by eNAD^+^ extraction and quantification using an Infinite 200 PRO (Tecan, Switzerland) microplate reader (11).

#### 2.5.1 Extraction of eNAD^+^ in plasma samples

Sample extraction was carried out as described previously (11). Briefly, an acid-heat extraction approach was used for heparinized plasma samples to extract eNAD^+^ following their collection from the −80°C freezer and subsequent thawing at room temperature. 300 μL of plasma were treated with 300 μL of 0.3 N HCl (Sigma-Aldrich, Catalog No: 339253) vortexed and then incubated at 60°C for 10 min. This was followed by sample equilibration on ice for 10 min and, finally, sample neutralization using 300 μL of neutralization buffer [0.36 N TEA-HCl (ACROS, United States, Catalog No: 170051000)] and 0.6 N KOH (Sigma-Aldrich, Catalog No: 484016) used in a 1: 1 ratio. Neutralized samples were centrifuged at 16,000 g for 10 min at 4°C and the supernatant was carefully pipetted on the assay plates for eNAD^+^ quantification.

#### 2.5.2 eNAD^+^ quantification

A two-step enzymatic reactant cycling assay with a colorimetric detection was used as previously described (11). Following sample extraction and neutralization, eNAD^+^ quantification in extracted and neutralized plasma samples was determined enzymatically using a master mix, β-NAD standard calibration solutions [S1 (0.753 μM) to S6 (0.023 μM)], and an Infinite 200 PRO (Tecan, Switzerland) microplate reader (11).

#### 2.5.3 Master mix solution

Briefly, a Master Mix (MM) solution contained all required molecules for the NAD^+^ cycling reaction, including Alcohol Dehydrogenase from yeast, EC1.1.1.1 [ADH, SIGMA, United States, Catalog No: A7011 (dissolved in Ammonium sulfate)], thiazolyl blue tetrazolium bromide (MTT, Sigma, United States, Catalog No: M2128), phenazine methosulfate (PMS, Sigma, United States, Catalog No: P9625), ethanol (100%), Triethanolamine (TEA, Sigma, United States, Catalog No: 90279), and diethyl dicarbonate (DEPC, Sigma, United States, Catalog No: D5758) water. TEA Buffer (pH = 7.4) and ADH preparation were diluted 10 fold with DEPC-H_2_O, while PMS and MTT solutions were prepared at 10 mg/mL and 1 mg/mL in DEPC-H_2_O, respectively. The MM was prepared fresh, directly prior to assay measurement.

#### 2.5.4 Assay measurement and plate reader settings

Samples, standard solutions from S1 to S6 and quality controls from QC1 to QC6 were loaded onto a 96-well plate. MM was prepared with MTT, PMS and ADH added directly before pipetting, where150 μL of the MM were pipetted into each well. Sample absorbance was measured at 565 nm in an Infinite 200 PRO (Tecan, Switzerland) microplate reader at 25°C. All measurements were performed with duplicates. No deoxygenation of neither solutions nor buffers was performed during any phase of the protocol at hand.

#### 2.5.5 β-NAD^+^ standard calibration solutions

Separate weightings of β-NAD (Sigma-Aldrich, Catalog No: N0632) were used to produce the primary stock solutions for the calibration curve standard solutions and eNAD^+^ quality controls. Primary stock solutions were prepared at a concentration of 1.50 μM using DEPC-treated deionized water (DEPC-H_2_O) and were stored at −80°C. Working solutions of β-NAD were prepared in DEPC-H_2_O using 3 times 1:10 dilution of the stock, to achieve a final dilution of 1:1000.

The first calibration standard sample (S1; 0.753 uM) was obtained by diluting 500 μL of the β-NAD working solution in 500 μL DEPC-H_2_O to reach a concentration of 0.753 uM. Five additional serial dilutions were prepared following the same procedure to obtain S2 (0.378 uM), S3 (0.177 uM), S4 (0.094 uM), S5 (0.047 uM), and S6 (0.023 uM).

The quality controls were created from separate working solutions using the same procedure as the calibration standard samples. A total of 6 quality controls were prepared corresponding to the same concentrations as the standard samples. All standard solutions and quality controls were aliquoted and stored at −80°C until use.

#### 2.5.6 NAD^+^ calibration curve

The calibration curve is constructed by the relative reaction velocity, v_R_, the first derivative of the absorbance over time (interval 5–25 min), on the Y-axis, and the theoretical standard concentration on the X-axis. The calibration curves were fitted using a linear regression created from the slopes of the standard solution [S1 (0.753 uM) to S6 (0.023 uM)] fitted through the origin (11). The equation of the linear regression was used to obtain the back-calculated concentrations of eNAD^+^ in plasma samples.

### 2.6 iNAD^+^ enzymatic assay

iNAD^+^ was measured using the iNAD^+^ enzymatic assay as previously described by Kanamori et al., with minor modifications (17). Briefly, 5 × 10^6^ freshly isolated PBMCs were used for the determination of iNAD^+^. Cells were pelleted using centrifugation at 500 *g* for 10 min. The cell pellets were washed with cold phosphate-buffered saline (PBS) followed by another centrifugation at 500 *g* for 10 min followed by cell counting using CASY cell counter (Omni Life Science, Catalog No: 5651736). The cell pellets were then extracted with 300 μL of 0.5 M perchloric acid and undergone 2 freeze–thaw cycle (snap frozen in liquid nitrogen and thawed in a 37°C heat block), followed by incubation on ice for 30 min, with 1 min-long vigorous vortexing every 5 min. The cells were then neutralized with 300 μL of the neutralization buffer (TEA: 0.55 M K^2^CO^3^ (1:1 ratio), Sigma, United States, Catalog No: 90279 and Catalog No: 367877). Samples were pH adjusted to fall within the 7.5–8.5 pH range using the neutralization buffer (17). Neutralized samples were centrifuged at 16,000 g for 10 min at 4°C and the supernatant was carefully pipetted on the assay plates for iNAD^+^ quantification.

#### 2.6.1 iNAD^+^ quantification

Following sample extraction and neutralization, iNAD^+^ quantification in cell pellet samples was determined following the same procedure as for the eNAD^+^ assay with minor modifications to the standards used [β-NAD standard calibration solutions: S1 (1 μM) to S6 (0.031 μM)]. The Infinite 200 PRO (Tecan, Switzerland) microplate reader was used for measurement. No deoxygenation of neither solutions nor buffers was performed during any phase of the protocol at hand.

#### 2.6.2 Calculation of iNAD^+^

The volume of a single cell is commonly referred to as the corpuscular volume. In a comprehensive study conducted by Simielie et al., involving over 500 clinical samples, the cell volume of PBMCs was carefully analyzed and determined to be 282.9 fl (18). Based on the findings, the concentrations of iNAD^+^ were subsequently calculated using the formula developed by Trammel and Brenner (19).

Therefore, the cell volume of 282.9 fl was multiplied by the total number of cells. Following that, the dilution factor was computed by dividing the volume used for extraction and neutralization by the total cell volume. Finally, the total iNAD^+^ concentration was determined by multiplying the dilution factor by the back-calculated concentrations derived from the standard curve.

### 2.7 Influence of variations in blood drawing and handling

#### 2.7.1 Cannula size

For the first stage, blood was drawn from four healthy volunteers using different cannula sizes (19 G, 21 G, 23 G and 25 G) (Braun, Venofix Safety: 4269071S-01, 4,056,504-01, 4,056,503-01, 4,056,502-01, and 4,056,501-01), keeping tourniquet time constant at 30 s.

#### 2.7.2 Tourniquet duration

A 21 G cannula was used to puncture the median cubital vein, followed by the application of a tourniquet for varying durations. Blood drawing was done at the end of each duration, including 0 s (without tourniquet), 30 s, 60 s, and 120 s.

#### 2.7.3 Storage time

Blood was drawn by puncture of the median cubital vein using a 21 G cannula with 30 s of tourniquet time. After blood collection, samples were stored for 0 min, 30 min, 60 min, and 120 min at 4°C followed by standard sample processing for eNAD^+^. Note, storage duration variations were followed by 15 min of centrifugation.

#### 2.7.4 Storage temperature

Blood was drawn using a 21 G cannula with 30 s of tourniquet time. Four different storage temperatures were examined (0°C, 4°C, 10°C, and 25°C) for 15 min each. Note, storage duration variations were followed by 15 min of centrifugation at each corresponding temperature.

### 2.8 Centrifugation settings

#### 2.8.1 Centrifugation duration

Blood was drawn by puncture of the median cubital vein using a 21 G cannula with 30 s of tourniquet. After blood collection, samples were placed in four different centrifuges and centrifuged at 2,500 g and 4°C for varying durations (5 min, 10 min, 15 min, and 25 min).

#### 2.8.2 Centrifugation speed

Blood was drawn using a 21 G cannula with 30 s of tourniquet. After blood collection, samples were processed according to standard sample processing with the respective variations to centrifugation speed (1,500 g, 2,000 g, 2,500 g, 3,000 g at 4°C for 15 min).

#### 2.8.3 Centrifugation deceleration mode

Blood was drawn using a 21 G cannula with 30 s of tourniquet. After blood collection, samples were centrifuged at 2,500 g at 4°C for 15 min followed by standard sample processing with the respective variations in the deceleration program. Deceleration modes differ in the braking profiles, with mode 9 having the fastest braking profile that causes the rotor to decelerate rapidly (30 s to full stop), mode 6 (4 min to full stop), mode 3 (12:15 min to full stop), and mode 1 (19:15 min to full stop) having the slowest braking profile causing the router to decelerate very slowly.

### 2.9 Statistical analysis

Statistical analysis was performed using GraphPad’s Prism 10 (GraphPad Software, La Jolla, CA, United States). A one-way analysis of variance (ANOVA – partition sum-of-squares), with repeated measures based on the general linear model, adjusted with Tukey’s multiple comparisons test, was used to evaluate the influence of the variations in blood sample collection (tourniquet time and cannula size) on the levels of eNAD^+^ in plasma. A Friedman test was used due to low sample size (with repeated measures, adjusted with Dunn’s multiple comparisons test) to evaluate the impact of the blood sample handling and pre-processing variations on the levels of eNAD^+^ in plasma. Overall, all tests were two-tailed and an alpha value of *p* < 0.05 was defined *a priori*. An unpaired, two-tailed Mann–Whitney U test was used to compare between the eNAD^+^ and iNAD^+^ in healthy, since the sample size was too small to pass normality tests. An unpaired, two-tailed *t*-test with Welch’s correction was used since the two datasets did not have equal standard deviations (SDs). A Pearsons correlation analysis was used, whereby the Pearson sample correlation coefficient (*r*) was used to establish the strength of correlation and an alpha value of *p* < 0.05 was defined *a priori*.

## 3 Results

### 3.1 Variance of eNAD^+^ and iNAD^+^

**Figure 1:**
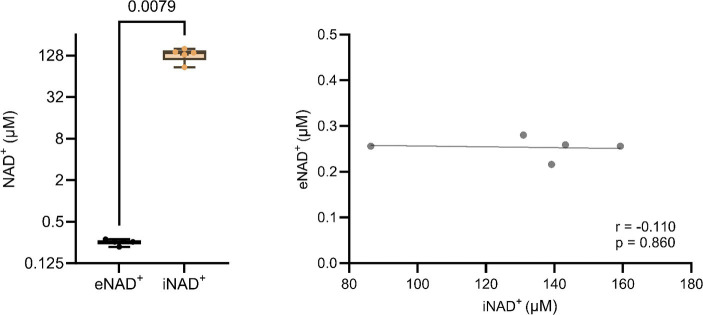
Difference in extracellular NAD^+^ in plasma and intracellular NAD^+^ levels in peripheral blood mononuclear cells (PBMCs) from **(A)** healthy volunteers (*n* = 5). **(B)** Pearsons correlation (*n* = 5) between PBMCs’ iNAD^+^ and plasma eNAD^+^. Statistics: **(A)** Statistical significance was determined using an unpaired two-tailed *t*-test. **(B)** Two tailed Pearson’s correlation coefficient was used with an alpha of *p* < 0.05.

At the outset, our primary objective was to ascertain the possible difference between eNAD^+^ and iNAD^+^, given their possible influence of iNAD^+^ on plasma measurements. Therefore, we measured NAD^+^ levels in both plasma and in PBMCs of healthy volunteers. There was a significant difference (Figure 1A), with healthy volunteers having approximately 520-fold higher iNAD^+^ concentration (131.8 ± 27.4 μM), when compared to eNAD^+^ (0.253 ± 0.02 μM, *p* = 0.0079). Next, we tested the correlation between the levels of eNAD^+^ and iNAD^+^ to detect any possible influences on eNAD^+^ from PBMCs’ iNAD^+^ (Figure 1B). However, we found no significant correlation between the levels of eNAD^+^ and PBMCs’ iNAD^+^ (*r* = −0.110; *p* = 0.860).

### 3.2 Impact of clinical laboratory parameters

**Figure 2:**
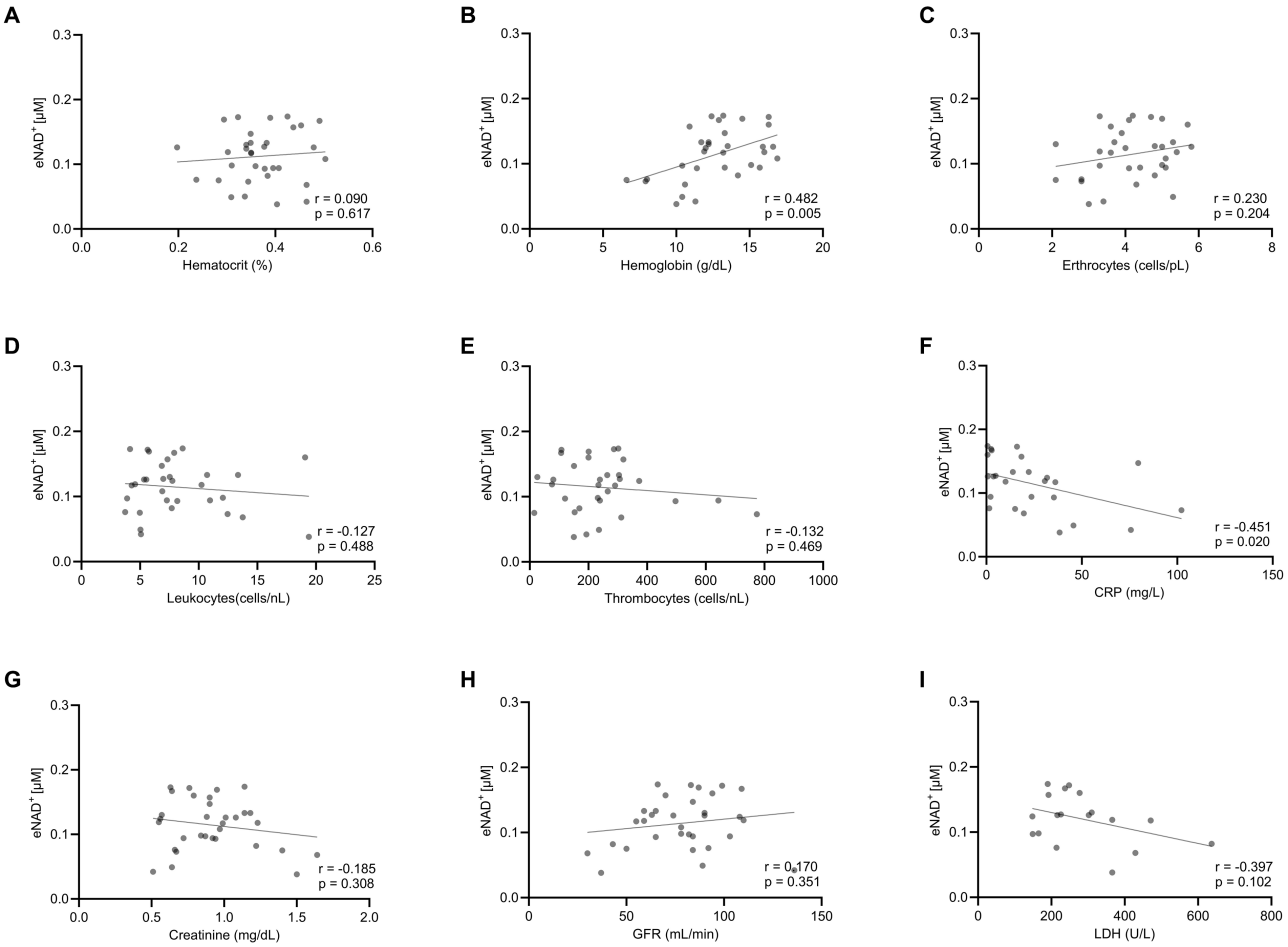
Correlation eNAD+ and clinical laboratory values of diseased patients (*n* = 33). eNAD+ levels were correlated to **(A)** Hematocrit, **(B)** Hemoglobin, **(C)** Erythrocytes, **(D)** Leukocytes, **(E)** Thrombocytes, **(F)** CRP, **(G)** Creatinine, **(H)** GFR, and **(I)** LDH. eNAD+ levels were significantly correlated with hemoglobin and CRP. (*p* = 0.005 & *p* = 0.020, respectively). Statistics: Two tailed Pearson’s correlation coefficient was used with an alpha of *p* < 0.05.

Moving forward, we tested potential interactions or correlations between routine clinical laboratory parameters and eNAD^+^ including hematocrit, hemoglobin, erythrocytes, leukocytes, thrombocytes, c-reactive protein (CRP), Creatinine, glomerular filtration rate (GFR), and lactate dehydrogenase (LDH) (Figure 2). Interestingly, eNAD^+^ levels were found to be in a statistically significant positive correlation with hemoglobin (*n* = 33; *r* = 0.482; *p* = 0.005). On the other hand, hematocrit, erythrocytes, leukocytes, and thrombocytes did not show any correlation with eNAD^+^ (*p* > 0.05). Furthermore, eNAD^+^ levels were found to be in a statistically significant negative correlation with CRP (*n* = 33; *r* = −0.451; *p* = 0.020), while there was no apparent correlation with renal function (GFR, creatinine). Of note, there was no observable correlation with LDH, a marker for necrosis (premature cell death) or apoptosis (programmed cell death) (*p* = 0.102). Collectively, hemoglobin appeared to be in a low positive correlation with eNAD^+^ levels while CRP levels were in a low negative correlation with eNAD^+^ levels.

### 3.3 Impact of blood drawing on eNAD^+^

**Figure 3:**
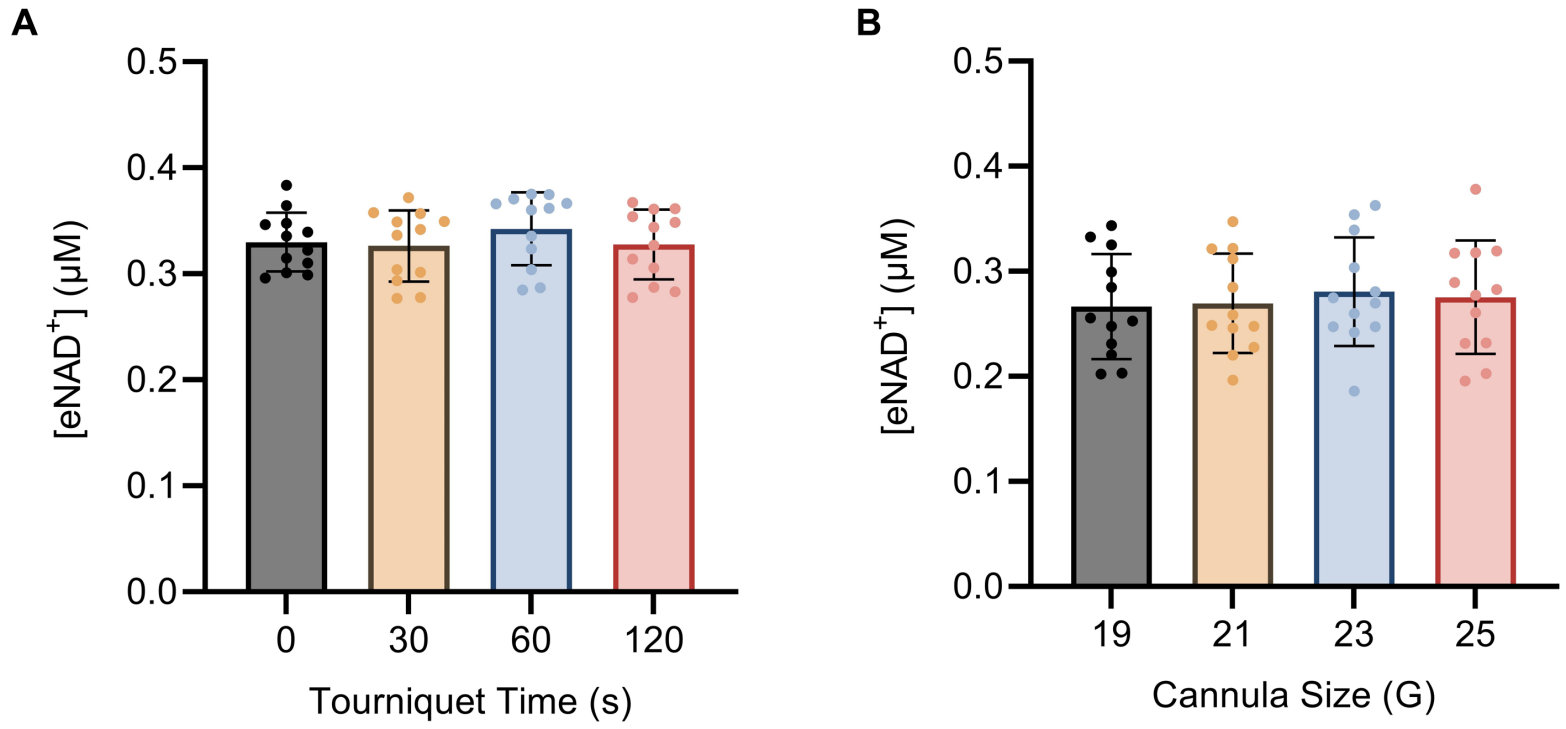
Impact of blood drawing with different **(A)** tourniquet times and **(B)** cannula sizes on eNAD^+^ (*n* = 4 healthy volunteers, four biological replicates and three technical replicates). No statistically significant difference in plasma eNAD^+^ concentrations was found. Statistics: One-way analysis of variance (ANOVA – partition sum-of-squares), with repeated measures based on the general linear model, adjusted with Tukey’s multiple comparisons test. Error bars represent standard deviation (SD) calculated from each group. Alpha of *p* < 0.05 was applied.

To investigate the impact of hemolysis on eNAD^+^ levels during blood drawing, we implemented changes to both the tourniquet time and cannula size. The shear stress exerted on the blood cells during the blood drawing can hypothetically induce cell lysis, which might result in the release of iNAD^+^. We tested four tourniquet time points (Figure 3A) and four cannula sizes (Figure 3B), but found no significant differences in measured eNAD^+^ for variations in either variable (*p* > 0.05). Notably, the tourniquet times and cannula sizes used in our study are consistent with routine clinical practices, with tourniquet times usually ranging from 30 to 60 s and 21 G cannulas being commonly used for venipuncture. Taken together, the variations in tourniquet times and cannula sizes did not appear to affect the level of measured eNAD^+^ in plasma samples.

### 3.4 Impact of sample handling on eNAD^+^

**Figure 4:**
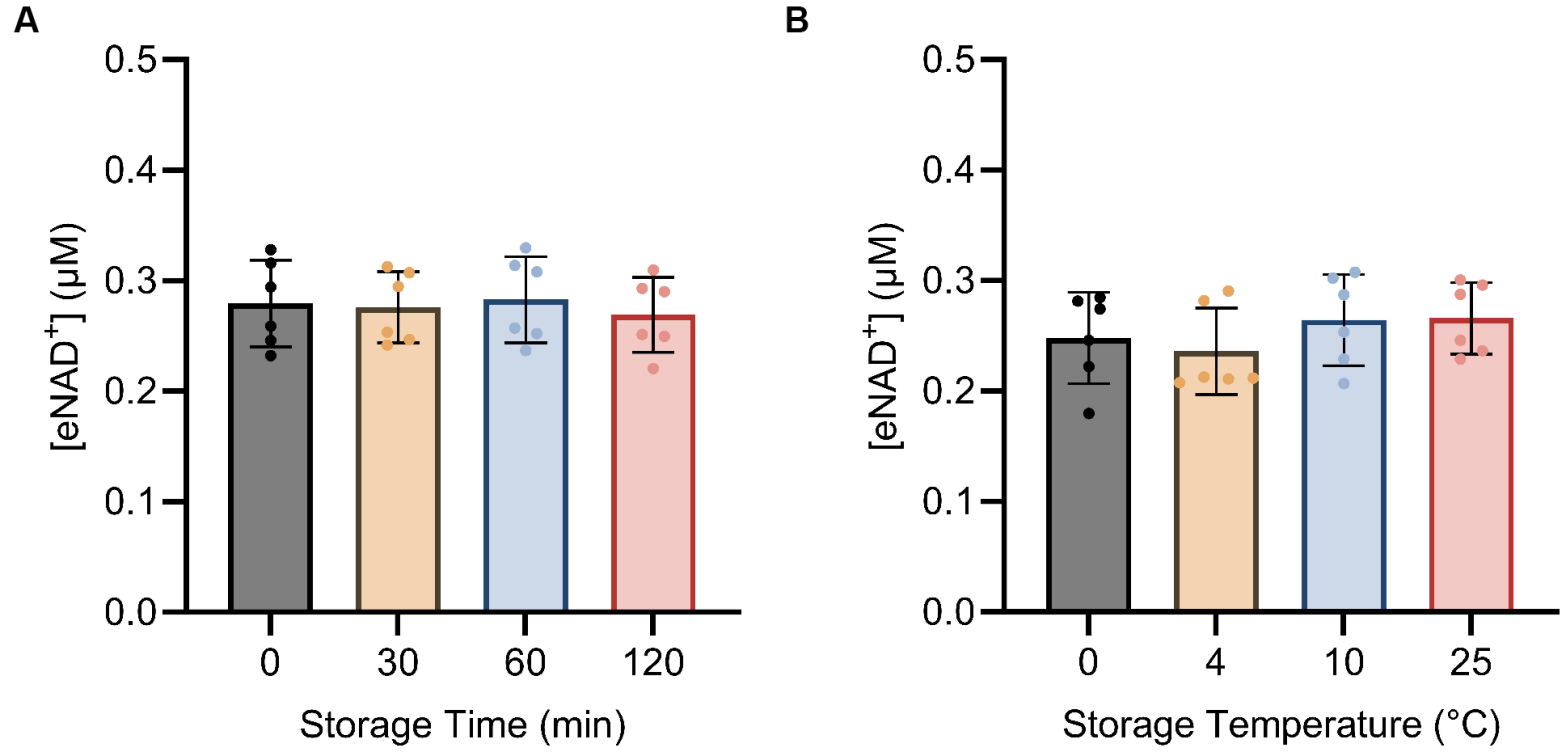
Impact of storage time **(A)** and storage temperature **(B)** on eNAD^+^ (*n* = 6 technical replicates). No statistically significant difference was found between any of the points for storage time and no significant difference was found between different storage temperatures for 15 min each. Note, storage duration variations were followed by 15 min of centrifugation at each corresponding temperature. Statistics: a Friedman test with repeated measures, adjusted with Dunn’s multiple comparisons test was used. Error bars represent standard deviation (SD) calculated from each group. Alpha of *p* < 0.05 was applied.

Next, we examined the impact of sample handling, time after blood drawing and temperature on eNAD^+^. Therefore eNAD^+^ in plasma was measured after different incubation times (Figure 4A) and temperatures (Figure 4B). Storage time and storage temperature appeared to have no significant effect on measured eNAD^+^ levels in plasma samples (*p* > 0.05), with storage time ranging from 0 to 120 min and storage temperature ranging from 0 to 25°C.

### 3.5 Influence of centrifugation parameters on eNAD^+^

**Figure 5:**
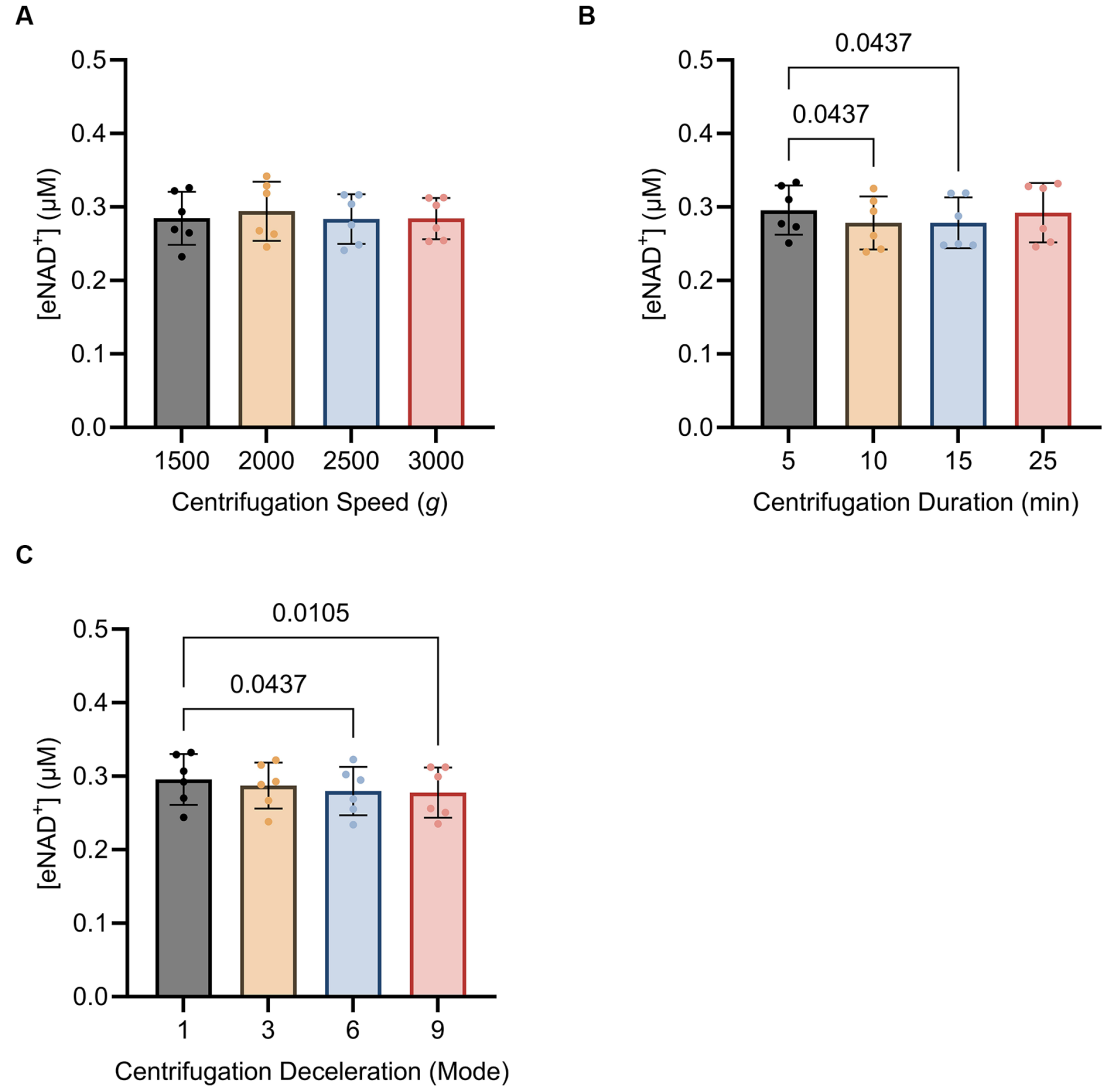
Impact of **(A)** centrifugation speed, **(B)** centrifugation duration and **(C)** centrifugation deceleration mode *n* = 6, technical replicates. Deceleration modes differ in the braking profiles, with mode 9 having the fastest braking profile that causes the rotor to decelerate rapidly (30 s to full stop), mode 6 (4 min to full stop), mode 3 (12,15 min to full stop), and mode 1 (19,15 min to full stop) having the slowest braking profile causing the router to decelerate very slowly. No statistically significant difference was found for the variations in centrifugation speed. A statistically significant difference was found for the variations in centrifugation duration between 5 min (0.295 ± 0.03 μM) and 10 min (0.278 ± 0.03 μM, *p* = 0.043) as well as 5 min (0.295 ± 0.03 μM) and 15 min (0.292 ± 0.04 μM, *p* = 0.043). A statistically significant difference was found between centrifugation mode 1 (0.295 ± 0.03 μM) and mode 9 (0.277 ± 0.03 μM; *p* = 0.010) and centrifugation mode 1 (0.295 ± 0.03 μM) and mode 6 (0.279 ± 0.03 μM; *p* = 0.043). Statistics: Statistics: A Friedman test with repeated measures, adjusted with Dunn’s multiple comparisons test was used. Error bars represent standard deviation (SD) calculated from each group. Alpha of *p* < 0.05 was applied.

Subsequently, we sought to determine whether centrifugation itself had any influence on eNAD^+^ levels, potentially resulting from cell lysis and shear stress. We found no significant difference (*p* > 0.05) between different centrifugation speeds (Figure 5A), ranging from 1,500 g to 3,000 g. Interestingly, for centrifugation duration (Figure 5B). eNAD^+^ decreased significantly by almost 6% with increasing centrifugation time. eNAD^+^ after 5 min was 0.295 ± 0.03 μM and after 10 min 0.278 ± 0.03 μM (*p* = 0.043). Next, we conducted tests on centrifugation deceleration modes (Figure 5C), wherein the durations the rotors took to come from high speed to a complete stop were varied. A faster deceleration phase yielded significantly lower eNAD^+^ levels. Deceleration mode 1 (19:15 min to full stop) demonstrated an average eNAD^+^ level of 0.295 ± 0.03 μM and mode 9 (30 s to full stop) an average of 0.277 ± 0.03 μM (*p* = 0.010), with a decrease of approximately 5%. Taken together, centrifugation duration and lower deceleration modes, with longer braking times, were found to reduce the measured eNAD^+^ in plasma samples. In contrast, centrifugation speed had no observable effect on measured eNAD^+^ in plasma samples.

## 4 Discussion

The interest in NAD^+^ research, specifically concerning the extracellular species, has reached unprecedented levels, leading to a substantial number of studies focusing on eNAD^+^ supplementation (20). Although this field is advancing rapidly, limited knowledge about quantitative measurement techniques hinders progress (21). Therefore, we have developed an enzymatic assay capable of quantitatively detecting plasma eNAD^+^ at nanomolar concentrations^11^. At the same time, iNAD^+^ and eNAD^+^ levels are supposed to vary significantly. The release of iNAD^+^ is consistently discussed as a potential source affecting extracellular measurements of eNAD^+^ (22). In this study we were able to quantify the differences between eNAD^+^ and iNAD^+^ levels and investigate possible impacts of laboratory parameters, and unraveled potential influences of blood drawing, sample handling on plasma eNAD^+^ levels. Furthermore, we present a first-hand protocol to accurately measure eNAD^+^ by implementing defined handling steps to avoid potential pitfalls.

Initially, we sought to discern the differences in concentration ranges between eNAD^+^ and iNAD^+^ (Figures 1A,B). Indeed, we discovered a noteworthy distinction between the levels of iNAD^+^ and eNAD^+^ in healthy volunteers’ PBMCs of approximate 500-fold difference (0.253 ± 0.02 μM vs. 131.8 ± 27.4 μM, *p* = 0.007, respectively). To the best of our knowledge, both eNAD^+^ and iNAD^+^ quantification for the same sample have not been done before in the literature. Reported concentrations of iNAD^+^ in healthy mammalian cells span a range of 25–500 μM [Erythrocytes ~25 μM (23); HeLa cells ~100 μM (19); HEK293 cells ~100–350 μM (24, 25); C2C12 myoblasts ~500 μM (26, 27); *Saccharomyces cerevisiae* ~400 μM (28); rat hepatocytes ~300 μM (28–30)]. In contrast, eNAD^+^ concentrations in plasma have been reported to fall within the range of 0.02–2.88 μM (7, 11, 31, 32), while they have utilized various semi-quantitative and quantitative methods, which may account for huge variations in the reported values. Our discovery of an ~500-fold difference in NAD^+^ levels between plasma and intracellular compartments NAD^+^ falls within the reported ranges, while both levels have been measured by a quantitative validated enzymatic assay (11).

The high concentration difference between iNAD^+^ and eNAD^+^ led us to investigate the potential correlation between iNAD^+^ and eNAD^+^, since the iNAD^+^ in PBMCs could potentially affect plasma eNAD^+^ during blood draw as a result of cell lysis. We found no significant correlation between PBMCs’ iNAD^+^ and eNAD^+^ (*p* = 0.860) in samples collected from the same individuals. Furthermore, our findings showed no statistically significant correlation between eNAD^+^ on any of the other blood cell types (Figure 2). This could indicate very minimal influence on eNAD^+^ from iNAD^+^ originating from erythrocytes, leukocytes, thrombocytes, or PBMCs’. Furthermore, that might suggest that the concentrations of iNAD^+^ and eNAD^+^ are not directly correlated and high eNAD^+^ does not necessitate high iNAD^+^ levels as well.

Next, we investigated the potential impact of the number of other blood parameters measured using laboratory parameters (Figure 2), on eNAD^+^ in patients. We found a statistically significant positive correlation whereby eNAD^+^ increased with increasing hemoglobin (*p* = 0.005). Surprisingly, hematocrit did not reach significant levels. Another interesting finding was the negative correlation of eNAD^+^ with CRP (*p* = 0.020). CRP as an inflammation marker is a critical component of the complement system and can be found to be elevated in various cancers and autoimmune conditions (33, 34). Lower levels of eNAD^+^ associated with higher CRP levels could be explained by the fact that, during an immune response, eNAD^+^ might be consumed at a higher rate by ectoenzymes like CD38, CD73, nucleotide pyrophosphatase/phosphodiesterase 1 and ADP-ribsoyltransferases (ARTS) on the cell membrane (9). As for the correlation between renal filtration parameters (GFR and creatinine) and eNAD^+^, we found no significant correlation (*p* > 0.5), however our findings were consistent with reported findings in the literature. We have found an increase in eNAD^+^ with increasing GFR and decreasing Creatinine, which confirms the findings from (35, 36) whereby, a decreased kidney function, potentially due to acute kidney injury, is followed by either a down surge of NAD^+^ biosynthesis or an elevated consumption of NAD^+^. Supplementation of NAD^+^ is discussed as a beneficial approach in cases of impaired kidney function (36). LDH has many assigned marker functions, especially when the condition is linked to cell death. In this context we analyzed the correlation between the LDH and eNAD^+^. However, despite the cells containing 500 times higher concentration of NAD^+^ than in plasma, we were surprised to find no correlation.

**Figure 6:**
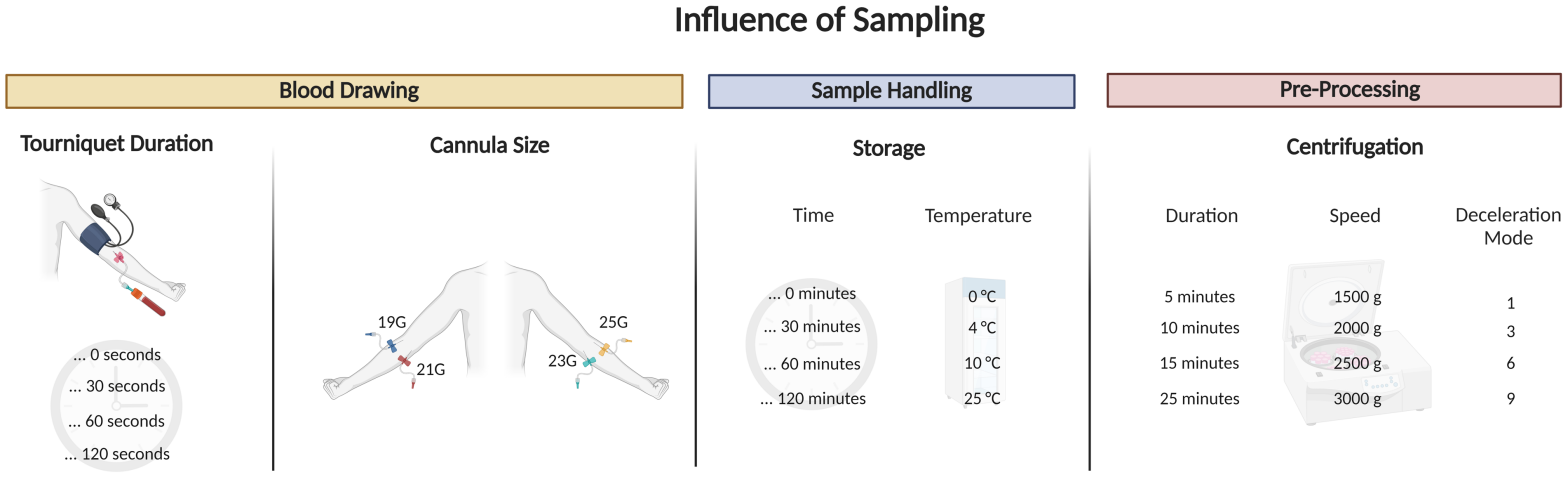
Graphical abstract outlining the blood draw trial that tested for different tourniquet durations, cannula sizes, sample handling and pre-processing tests on extracellular NAD^+^.

After investigating the correlations between eNAD^+^ levels and various blood parameters, we proceeded to examine the potential influence of blood drawing, sample handling and pre-processing steps on the levels of eNAD^+^ (Figure 6). Since eNAD^+^ measurements in bodily fluids like plasma or serum may be significantly impacted by cell lysis (37); hemolysis which can occur directly from blood drawing and cause cell lysis, *in vitro*, through the lytic release. Thus iNAD^+^ could be a significant contributor to altered eNAD^+^ levels. This distinction must be made from *in vivo* hemolysis, which can occur independently of any blood collection (38), and was not investigated in this experiment. On the other hand, *in vitro* hemolysis might be caused by inadequate specimen collection or handling (38). Therefore, we explored the effects of several tourniquet durations and cannula sizes in healthy volunteers to see if this had an impact on the measured eNAD^+^ levels (Figure 3). Our findings showed no significant changes in measured eNAD^+^ levels with tourniquet durations between 0 to 120 s. These tourniquet times are within a typical clinical range. In a recent observational study by Jacob et al. involving over 300 patients, various parameters associated with hemolysis were investigated (39). The authors concluded that hemolysis increased by 22% with every minute of tourniquet application. However, their analysis lacked a detailed description of the patients and did not specify their underlying diseases, only providing the region of diagnosis (e.g., abdomen). This raises the question of whether their findings are true or are merely impacted by surrogate parameters like the sickness of the patients. For instance, a longer tourniquet time can be necessary due to dehydration, low blood pressure, fragile veins, hard-to-find veins and the greater difficulties in obtaining venous access in the elderly (40, 41). At the same time, it is worth noting that sicker patients have increasing hemolysis, for other reasons than the tourniquet time itself (42, 43). Hemolysis can be increased due to infections, sepsis or surgery (39, 44). However, this information has been neglected in the mentioned paper. In contrast, our blood drawing trial included only healthy volunteers, and we excluded all potential pathological factors like infection or previous interventions or surgeries.

Next, we tested the effect of different cannula gauges during venipuncture on the levels of measured eNAD^+^. We observed no statistically significant difference in measured eNAD^+^ between the four different cannula sizes employed (19 to 24 G). In light of the evidence presented, we found no conclusive evidence to support the presence of an effect caused by variations in blood sample drawing that significantly impacted the measured eNAD^+^ levels in plasma. This is in line to a randomized trial in emergency patients on hemolysis and using intravenous catheters versus venipuncture tubes (45). The researchers demonstrated when using Vacutainer venipuncture, no significant hemolysis was observed, whereas blood samples obtained through an IV-catheter exhibited notably higher levels of hemolysis. We used a similar Vacutainer venipuncture method to draw blood in our blood drawing trial. In conclusion, with the variations of different tourniquet times and cannula sizes, we reject the hypothesis that the tourniquet time and cannula size may affect the measured eNAD^+^ in plasma samples with the tested set up.

Thereafter, we investigated the effect of sample handling, storage time and temperatures on measured plasma eNAD^+^ concentrations (Figure 4). Our findings showed that plasma eNAD^+^ levels were neither affected by variations in storage times of up to 120 min at 4°C, nor by storage temperatures ranging from 0°C to 25°C for 15 min. Moving on to the sample pre-processing step, the centrifugation speed, centrifugation time, and centrifugation deceleration modes were tested for their impact on the measured eNAD^+^ concentrations (Figure 5). For the variations in the centrifugation speed, we found no statistically significant difference in eNAD^+^ concentrations (*p* > 0.05) This is consistent with recent findings from Allison et al., where they have found that the quality of the plasma (no significant effect on plasma metabolites) was not affected by centrifugation speeds of 1,000, 2,000, 3,000, and 4,000 RPM within a time from 3 to 15 min, for tested analytes sodium, potassium, and bicarbonate (46). Interestingly, measured eNAD^+^ levels were higher at the 5 min and 25 min centrifugation durations (0.295 ± 0.03 uM and 0.292 ± 0.04 uM, respectively) with a statistically significant difference with lower centrifugation times (*p* < 0.05). The observed differences remain vague but might be attributed to the reduced separation efficiency of cellular constituents with shorter centrifugation time (5 min) (47). Hemolysis and/or structural damage might occur with prolonged centrifugation time (25 min) potentially leading to plasma contamination with intracellular substances, such as iNAD^+^ and other enzymes like ADP-ribosyl transferases, PARPs, and Sirtuins, which are known to be released as a result of cell damage (48). Some of which may interact with the components of the master mix used in the assay or NAD^+^ or NADH, directly or indirectly and could falsely increase the measured levels of eNAD^+^. Taken together, our findings suggest that a centrifugation of 10–15 min is recommended for optimal separation without risking falsely high or low NAD^+^ levels.

Lastly, the centrifugation deceleration mode variations were investigated in the sample pre-processing step. Centrifugation deceleration mode refers to the strength of the brake and consequently the time the centrifuge comes to a full stop after a complete spin cycle. A high deceleration mode (mode 9) brings the rotor to a stop rapidly, while a low deceleration mode (mode 1) allows the rotor to stop slowly. Our findings suggest a decreasing trend noticeable where the highest measured eNAD^+^ levels were found with deceleration mode 1 (long time to full stop; ~19 min) and the lowest with deceleration mode 9 (short time; ~30 s). The deceleration mode exhibited a distinct impact on eNAD^+^ measurements. Gentle deceleration resulted in a higher eNAD^+^ concentration compared to mode 9’s abrupt stop. A plausible explanation is the possibility of remixing of the cells with plasma layers when applying the abrupt stop. This finding conforms with the recommendations from International Council for Standardization in Hematology (ICSH) to avoid rapid deceleration at the end of centrifugation to prevent remixing of cells and plasma around the plasma/buffy coat interface (49). Therefore, they recommend gentle centrifuge braking and use of swing out rotors to help prevent remixing.

One of the limitations of the current study is the relatively small sample size in the blood drawing trial, which was, however, in accordance with ethical approval. A major caveat in NAD^+^ quantification is that the accuracy of the measurements, whether with LC/MS or enzymatic setups, is highly dependent on the extraction protocol. NAD^+^ and NADH stability outside of the cellular environment, particularly in biofluids and during storage and processing for measurements, might be influenced by temperature, pH, time and other factors. NAD^+^ is generally more stable than NADH. It is quite resistant to degradation in a wide range of pH levels, but decomposes rapidly in alkaline solution (50). Acidic conditions can help to preserve the integrity of NAD+ because the molecule is less prone to hydrolysis and breakdown in such an environment. On the other hand, NADH is prone to autoxidation, and degrades rapidly in acidic solutions into a non-NAD^+^ species that is not detected by our assay. As such rapid sample quenching and extraction is crucial for accurate measurements of NAD(H) (23).

In our experimental setup, quick extraction is essential to minimize the enzymatic reactions that might alter eNAD^+^ and NADH levels. Enzymes present in the plasma might catalyze the degradation or interconversion of these molecules. Furthermore, acid extraction of NAD^+^ is favorable because the low pH can minimize the risk of any enzymatic activity that could otherwise alter NAD^+^ levels from enzymes present in the samples during or after extraction. The acid stabilizes the NAD^+^ by preventing its conversion to NADH by eliminating the presence of any biocatalysts, which could skew quantification results.

Another important influencing factor on NAD (H) is the effect of oxygen, since NADH is particularly sensitive to oxidation (51). At physiological pH, NADH was found to be unstable in the presence of oxygen and gave rise to an NADH-peroxide that could serve as a source of H_2_O_2_ (52). Furthermore, in longer extraction protocols, there is a higher risk of enzymatic degradation and oxidation, particularly for NADH. This is due to the reactive nature of NADH, which can donate electrons and convert to NAD^+^ upon exposure to oxidative conditions over time. The use of degassed buffers and media could contribute in reducing the oxidation risk by removing dissolved oxygen, which might partially protect NADH from oxidation to NAD^+^.

Another limitation might be the possibility of oxidation of NADH to NAD^+^, which might affect NAD^+^ measurements and impact the accuracy of the measurement in biological samples as metabolite degradation and interconversion may occur during extraction and after the extract is made (53). However, the use of deoxygenated media and buffers may minimize this effect.

Another limitation when evaluating the comparability of this study, is the lack of a gold standard when it comes to eNAD^+^ quantification in plasma. While LC/MS methods are generally considered as such in analytical chemistry, there seems to be an apparent need for further optimization of LC/MS setups when it comes to reliably measuring a eNAD^+^ in human plasma. This may be explained by appreciating the complexities inherent with NAD^+^ measurements, including the instability of NAD^+^, the potential for interconversion between NAD^+^ and NADH, and metabolite degradation during and post-extraction, particularly under typical LC/MS conditions (53). In fact, these become even more pronounced at the very low concentrations of NAD^+^ commonly found in blood plasma. Furthermore methodological limitations such as matrix effects and variations in ionization efficiency, can lead to ion suppression and, consequently, inaccuracies in absolute quantification. These complexities necessitate thorough optimization of LC/MS methods to produce reliable results for plasma eNAD^+^ quantification (54). Yet, it is important to note that while LC/MS has been a robust and validated technique for NAD^+^ measurements in whole blood, correlating well with enzymatic assays (55–57), such an equivalence has yet to be established for blood plasma. Nevertheless, the data is novel, and the inclusion of healthy volunteers has ruled out surrogate parameters of age or disease, which is the case for many other studies. Further research on eNAD^+^ demands validation data, and we are confident that our findings contribute significantly to the NAD^+^ research area.

## 5 Conclusion

This study aimed to investigate the differences between eNAD^+^ and iNAD^+^ levels and examine the potential influences of blood drawing, sample handling and pre-analytical factors on plasma eNAD^+^ levels and to shed light on possible correlations with laboratory parameters. Remarkably, our study uncovered that eNAD^+^ levels were approximately 500 times lower than the levels observed in PBMCs. However, we did not find any significant correlation between eNAD^+^ and iNAD^+^, nor with the different cell types in the blood. Notably, variations in blood drawing, such as tourniquet duration and cannula size, as well as sample handling, including storage duration and temperature, did not have a significant impact on eNAD^+^ levels. On the other hand, the sample pre-processing steps, specifically centrifugation duration and deceleration mode, exhibited statistically significant effects on plasma eNAD^+^ levels.

eNAD^+^ quantification is an intricate process and the impact of extraction protocols cannot be overstated. As such, it is imperative to consider the effect of factors such as pH, time, and the presence of oxygen in the extraction process, whether using LC/MS or enzymatic setups. To facilitate future investigations into eNAD^+^, we have developed and provided an optimized easy to follow protocol for quantifying plasma eNAD^+^ from blood samples, encompassing the entire process from drawing the samples to sample processing. This will help to overcome existing challenges and ensure the reliability findings while delving deeper into the realm of eNAD^+^ research.

## Data availability statement

The raw data supporting the conclusions of this article will be made available by the authors, without undue reservation.

## Ethics statement

The studies involving humans were approved by Ethikkommission der Charité Universitätsmedizin Berlin (EA2/159/22, EA1/291/16 and EA1/096/18). The studies were conducted in accordance with the local legislation and institutional requirements. The participants provided their written informed consent to participate in this study.

## Author contributions

AS: Conceptualization, Investigation, Resources, Writing – original draft, Writing – review & editing, Data curation, Formal analysis, Methodology, Validation, Visualization. CK: Conceptualization, Data curation, Investigation, Resources, Writing – original draft, Writing – review & editing, Data curation, Formal analysis, Investigation, Methodology, Resources, Validation, Visualization, Writing – original draft, Writing – review & editing. PB: Conceptualization, Resources, Writing – review & editing, Funding acquisition, Project administration. NH: Visualization, Writing – original draft. PK: Visualization, Writing – original draft. K-HH: Visualization, Writing – original draft. EK: Writing – review & editing. SM: Writing – review & editing. RM: Writing – review & editing. NR: Writing – review & editing. JP: Writing – review & editing, Supervision. FK: Supervision, Writing – review & editing, Conceptualization, Funding acquisition, Investigation, Project administration, Resources, Writing – original draft.
