# Supplementary material for: Optimized protocol for quantification of extracellular nicotinamide adenine dinucleotide: evaluating clinical parameters and pre-analytical factors for translational research
Source: Front Med (Lausanne). 2024 Jan 8;10:1278641. doi: 10.3389/fmed.2023.1278641 (PMC10800990; doi:10.3389/fmed.2023.1278641)
Supplement: Supplementary file 1 [file Table_1.DOCX]

Optimized Protocol for Quantification of Extracellular Nicotinamide Adenine Dinucleotide (eNAD+) in Plasma: Evaluating Clinical Correlations and Pre-analytical Factors for Clinical and Translational Research

# Supplementary Protocol: eNAD+ Extraction and Quantification in Plasma SOP

# The purpose of this standard operation procedure (SOP) is to outline the procedure for extraction and quantification of extracellular Nicotinamide Adenine Dinucleotide (eNAD^+^) in plasma samples.

| Reagent | Components | Catalog No. |
| --- | --- | --- |
| Master Mix  To be prepared by the user just before use | Alcohol Dehydrogenase (ADH): 1:10 dilution of ADH Solution in DEPC-Water | A7011 |
|  | Triethanolamine (TEA): 1:10 dilution of TEA in DEPC-Water, pH 7.4 | 90279 |
|  | Ethanol (EtOH): 100% | 51976 |
|  | Phenazine methosulfate (PMS): 10 mg/mL solution | P9625 |
|  | 3-(4,5-dimethylthiazol-2-yl)-2,5-diphenyltetrazolium bromide (MTT): 1 mg/mL solution | M2128 |
| Standard Matrix Solution  To be mixed by the user, aliquoted and stored at -20°C | Sodium chloride (NaCl) |  |
|  | Sodium bicarbonate (NaHCO3) |  |
|  | Sodium carbonate (Na2CO3) |  |
|  | Potassium chloride (KCl) |  |
|  | Monopotassium phosphate (KH2PO4) |  |
|  | Magnesium chloride hexahydrate (MgCl2•6H2O) |  |
|  | 2-[4-(2-hydroxyethyl)piperazin-1-yl] ethane sulfonic acid (HEPES) |  |
|  | Calcium chloride dihydrate (CaCl2•2H2O) |  |
|  | Sodium sulfate (Na2SO4) |  |
|  | Bovine serum albumin (BSA) |  |
| NAD^+^ Standard | Nicotinamide adenine dinucleotide (β-NAD) | N0632 |
| NAD^+^ Extraction buffer | 0.3N HCl | 339253 |
| NAD^+^ Neutralization buffer | 0.36N TEA-HCl: 0.6N KOH (ratio 1:1) | 90350, 221473 |

# Procedure:

1. Blood Collection
   1. Use a 21 G cannula to collect blood into heparin tubes.
2. Sample Processing
   1. Centrifuge blood samples at 2500 *g* for 15 minutes at 4 °C to separate corpuscular blood components from plasma.
   2. Aliquot plasma, snap freeze, and store at -80 °C.
3. Sample Preparation:
   1. Retrieve heparin plasma samples from -80°C freezer.
   2. Allow the samples to thaw at room temperature (Note: NAD^+^ is stable for up to 30 minutes at room temperature).
4. eNAD+ Extraction:
   1. Transfer 300μL of each thawed plasma sample into a fresh tube.
   2. Add 300 μL of 0.3N HCl to each tube, vortex, and incubate at 60 °C for 10 min.
   3. Equilibration:
      1. Cool samples on ice for 10 min.
   4. Neutralization (Neutralize the samples to achieve the optimal working pH of 7.4 for ADH):
      1. Neutralize samples with 300 μL of neutralization buffer (1:1 0.36N TEA-HCl and 0.6N KOH).
   5. Deproteinization:
      1. Centrifuge samples at 16,000 g for 10 min at 4 °C to remove any proteins.. Sample Loading:
   6. Sample loading:
      1. Carefully load 50 μL the clear supernatant without disturbing the pellet of each sample into the wells of a 96-well plate.
5. eNAD^+^ Quantification
   1. Prepare the Master Mix (MM) solution containing TEA (diluted 1:10 with DEPC-H_2_O), DEPC-H_2_O, EtOH, PMS, MTT, and ADH (diluted 1:10 with DEPC-H_2_O) in this order. Prepare the master mix directly before assay measurement.
   2. Load 50 µL of r-SBFA into the corresponding wells for standards and Quality controls and 50 µL into the corresponding wells for samples.
   3. Load standard solutions (S1 (0.753 µM) to S6 (0.0235 µM)), Quality Controls (QCs), and samples onto appropriate wells.
   4. Add 150 μL of the MM into each well.
   5. Measure sample absorbance at 565 nm in the microplate reader at 25 °C. Perform all measurements in duplicate and repeat assay runs at least 3 times.
6. NAD^+^ Calibration Curve
   1. Plot the first derivative of absorbance (vR) against the theoretical standard concentration.
   2. Use a linear regression line through the origin for the calibration curve.
   3. Use the equation from the linear regression to calculate the eNAD^+^ concentration in plasma samples.
7. Preparation of β-NAD^+^ Standard Calibration Solutions
   1. Prepare primary stock solutions for calibration curve standard samples (S1-S6) and eNAD^+^ quality control samples (QC1-QC6) using a concentration of 1 µg/mL.
   2. Obtain S1 by diluting 500 µL of β-NAD working stock solution in 500 µL DEPC-H_2_O. Prepare five additional serial dilutions for S2-S6.
   3. Prepare quality control samples using the same procedure.

|  | NAD^+^ Concentration (µM) |
| --- | --- |
| S1 / QC1 | 0.7536 |
| S2 / QC2 | 0.3768 |
| S3 / QC3 | 0.1884 |
| S4 / QC4 | 0.0942 |
| S5 / QC5 | 0.0471 |
| S6 / QC6 | 0.02355 |

Storage:

Store the extracted NAD^+^ samples at -80°C for long-term storage.
